# Specialised placement of morphs within the gall of the social aphid *Pemphigus spyrothecae*

**DOI:** 10.1186/1471-2148-7-18

**Published:** 2007-02-13

**Authors:** Nathan Pike

**Affiliations:** 1 Department of Zoology, University of Oxford, South Parks Road, Oxford OX1 3PS, UK

## Abstract

**Background:**

The strategic placement and aggregation of certain castes within the nests of eusocial insects such as bees and ants results in complex colonies that enjoy increased fitness through improved efficiency of social tasks. To examine if this advanced social phenomenon might apply to social aphids, the location of the numerous morphs within the nests (plant galls) of the aphid species *Pemphigus spyrothecae *was examined.

**Results:**

A strong, almost exclusive tendency for soldiers to occupy the gall section nearest to the opening was detected. In addition, it was found that the most reproductively valuable morphs, the mature gall reproductives and the adult gall emigrants, tended to locate themselves in the gall section furthest from the opening.

**Conclusion:**

The defensive morphs are thus ideally placed at the point most vulnerable to predation while the morphs most directly responsible for the colony's fitness are located in the safest area of the nest. Furthermore, the propensity for soldiers alone to be located at the opening provides excellent supporting evidence that they are also the agents of gall cleaning and repair. These observations demonstrate that relatively high levels of spatial complexity can occur within the galling colonies of the social aphids, just as they occur within the advanced societies of other insect orders.

## Background

In many aphid societies, in addition to the renowned phenomenon of defence by morphologically specialised or unspecialised individuals, a broad range of social tasks can be found. Social aphids may clean their nests of honeydew and detritus [1-4], undertake risky migration to other colonies [5,6], and repair damage to the plant galls they induce to serve as their nests. However, the clustering and specialised placement of castes that is a celebrated adaptive phenomenon of better-studied social insects such as the ants [7,8] and bees [9] is currently unknown in the social aphids. It is nevertheless clear that, because the number of soldiers in an aphid colony may scale more accurately with gall surface area than with the number of non-soldiers needing defence [10], gall structure can be of critical importance to the evolution of aphid life histories. Detection of any non-random distribution of castes within aphid galls would indicate a greater level of social complexity than that which is currently appreciated.

Spatial distributions which involve certain aphid morphs clustering near the gall openings are important for their likely relevance to gall repair. In the case of the spectacular, speedy and suicidal nest repair seen in *Nipponaphis monzeni *[11], it is evident from observations that it is the defensive individuals, the soldiers, that are responsible for exploding their bodies to release a sticky plaster into which they embed themselves as it hardens to reseal their gall. However, in other species which repair their gall more slowly by inducing complementary regrowth from an undamaged area of the gall, evidence for preferential clustering of soldiers or other morphs at gall openings (that potentially require repair) has not been sought.

Soldiers of *Pemphigus spyrothecae*, the study animal of the current research, are known to repair their gall through such complementary regrowth [12]. The species forms spiral galls on the petioles of poplar leaves at budburst and these galls remain tightly closed for two to three months until the aphids create a small hole, the ostiole, through which the winged migrant adult morphs exit [13]. It has been empirically demonstrated that the ostiole and other gall openings caused by damage are weak points that increase rates of predation [12].

Because the morphologically specialised soldiers of *P. spyrothecae *are best able to deal with invading predators, one might reasonably hypothesise a tendency for soldiers to gather near the gall opening while non-soldiers avoid this area. Aside from indicating a hitherto unknown level of colony complexity, such a tendency would represent further support for the evidence provided by Pike and Foster [12] that there is strong division of labour in the gall repair task and that soldiers are the predominant agents of this repair. There is also reason to expect soldiers to predominate near the ostiole because it is the aperture through which they, as the primary cleaners, perform their essential waste dumping duties [3]. The general goal of the current research was thus to assess whether any non-random distribution of aphid morphs occurs within the gall of *P. spyrothecae*.

## Results

Three of the 20 galls were subject to catastrophic predation that resulted in death of all aphids. In only one of these cases was the predator (a third-instar hoverfly larva, Syrphidae: Neuroptera) still present. These three galls that were devoid of living aphids were excluded from further analyses. Predators were also found in nine of the remaining 17 galls. All nine of these galls contained hoverfly larvae, with a mean (± SE) of 1.78 ± 0.56 being found in each of these galls. Most hoverfly larvae (13/16) were in their first instar but a second-instar hoverfly larva was found in three separate galls. A first-instar lacewing larva (Chrysopidae: Neuroptera) was also found in one of the nine galls that were subject to hoverfly predation. The effect of these predators on the population and demographic composition of the galls was not found to be significant (ANOVAs for predation effect on population size and morph proportions: *P *> 0.35 in all cases) and, for this reason, the nine galls containing predators were pooled with the eight galls devoid of predators in further analyses.

**Figure 1:**
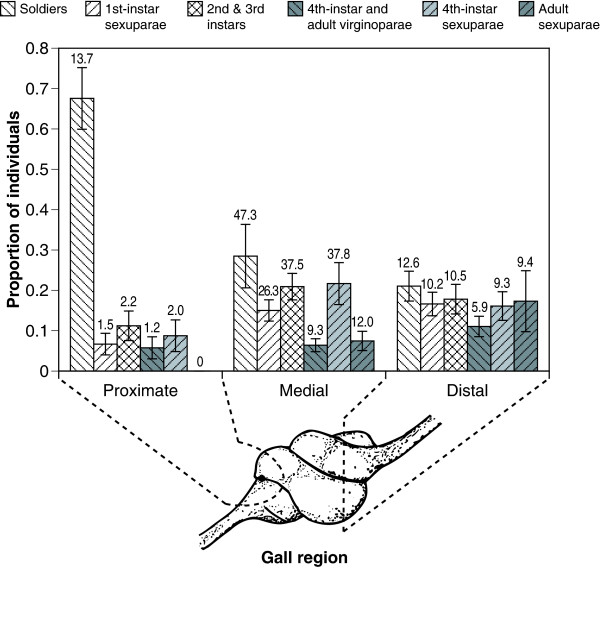
**The average proportion of aphids in each of the six morphological classes**. Data (mean proportions ± 95% C.I.) are plotted separately for the three gall sections that are proximate, medial or distal to the gall's opening. The actual numbers of individuals are given as numerical values (means) above each column.

For none of the six morphological classes did the proportionate representation in the population differ significantly among galls (e.g. for soldier proportion, F_16,34 _= 0.28, *P *= 0.996). This factor of gall was thus statistically eliminated in the subsequent analyses that compared morph proportions by gall section. The average proportions (and numbers) of each aphid morph in each gall section are shown in Figure 1.

The proportion of soldiers differed significantly among gall sections (F_2,48 _= 46.48, *P *< 0.001). Tukey's HSD post-hoc contrasts indicated that the proportion of soldiers in the gall section proximate to the ostiole (0.676 ± 0.009) is significantly higher than the proportion of soldiers in either the medial or the distal sections (respectively, 0.285 ± 0.009 and 0.211 ± 0.004, *P *< 0.001 in both cases). No difference in soldier proportion was detectable between the medial and distal sections (*P *= 0.34).

After having accounted for this section-based difference in soldier proportion, another significant section-based difference in the proportion of adult sexuparae (winged migrants from gall) was found to persist. Aside from being obviously greater in the non-proximate gall sections, the proportion of adult sexuparae in the gall section distal to the ostiole (0.173 ± 0.009) was found to be significantly greater than in the medial section (0.075 ± 0.003) (Tukey HSD, *P *= 0.003). Similarly, a Tukey HSD test found that the greater proportion of fourth-instar and adult virginoparae (gall reproductives) in the distal section (0.111 ± 0.003) than in the medial section (0.064 ± 0.003) was marginally significant (*P *= 0.049).

## Discussion

There is a strong bias for soldiers rather than other morphs to occupy the section of the gall immediately around the ostiole. Based on the assumption that the soldiers of *P. spyrothecae *will cluster around artificial holes just as they cluster around natural ostioles, there is excellent supporting evidence that these soldiers are the predominant agents not only of defence and cleaning, but also of nest repair. Recent speculation [12,14] about the breadth of altruistic behaviours by soldiers of this species is thus supported. Multiple altruistic behaviours can also be expected in other aphid species. It is relevant that, although defence appears to be the most widespread and obvious of these behaviours, it need not feature in altruists' repertoire: we know, for example, that the first instars of *Hormaphis betulae*, a species without soldiers, co-operate to push large masses of honeydew out of their galls [2].

The stratified distribution of adult gall emigrants and maturing/mature adult gall reproductives is remarkable. It is clear that the adult sexuparae tend to aggregate in the area of the gall which we can assume to be the most protected, as it is the most distant from the opening. An identical preference is present in the fourth-instar and adult virginoparae. It is likely to be no co-incidence that the mature individuals of the morphological classes (which have the highest reproductive value) inhabit the safest region of the gall.

## Conclusion

The evolutionary process that has facilitated aphid sociality by selecting for group co-operation in exploiting and protecting the rare and valuable gall habitat has been called 'fortress defence' [15]. It is intriguing that, even within the gall fortress, there appears to be a region that is a relative stronghold which is optimally exploited through non-random placement of gall inhabitants. In quantifying this non-random placement, the current study provides the first demonstration of the potential for spatial complexity within the galling colonies of social aphids to be considerably greater than previously thought.

## Methods

20 galls of *Pemphigus spyrothecae *were collected on 7 August 2005. Natural ostioles had formed in these galls between one and two months earlier. Two galls were collected from each of ten Lombardy poplar trees, *Populus nigra*, located at a field site (51° 43' 58" N, 1° 13' 0" W) in Oxford, UK. Immediately that the galls were removed from the tree (i.e. before the aphids had opportunity to move), they were cut into the following three sections: (1) the section proximate to and surrounding the gall's ostiole, being located within 3 mm of this opening, (2) the medial section which began at the cut point of the first section and spanned up to the final section, (3) the 5 mm section of the gall that was most distal to the ostiole. Each of these sections was then immediately preserved in 80% ethanol in a separate vial along with all aphids it contained. A diagram of a gall indicating the three sections is included in Figure 1.

The contents of each section were examined through a dissecting microscope and the exact numbers of individuals in each of the following six morphological classes were recorded: (1) first-instar virginoparae (soldiers), (2) first-instar sexuparae, (3) second and third instar virginoparae and sexuparae, (4) fourth-instar and adult virginoparae, (5) fourth-instar sexuparae, and (6) adult sexuparae. (The instars of the virginoparous morph are the sedentary gall inhabitants while the instars of the sexuparaous morphs are the pending emigrants.) Galls were also examined for the presence of predators which, if immature, were identified to at least family level.

Numerical analyses were largely based on comparing differences among gall sections in the proportion of the aphid population made up by a focal morph. To ensure conformity with assumptions of normality, these proportion-based data were arcsine square root transformed.

## Authors' contributions

N. P. designed the research, conducted the observations, analysed the data, and wrote the manuscript.
